# Immature Honey as a Quality Challenge in Global Apicultural Production

**DOI:** 10.3390/foods15122136

**Published:** 2026-06-13

**Authors:** Anna Gajda, Bartosz Lewandowski, Przemysław Rujna, Joanna Katarzyna Banach, Renata Pietrzak-Fiećko, Ewaryst Tkacz

**Affiliations:** 1 Laboratory of Bee Diseases, Institute of Veterinary Medicine, Warsaw University of Life Sciences, 02-787 Warsaw, Poland; anna_gajda@sggw.edu.pl; 2 Department of Clinical Engineering, Academy of Silesia in Katowice, 40-555 Katowice, Poland; bartosz.lewandowski@akademiaslaska.pl (B.L.); ewaryst.tkacz@akademiaslaska.pl (E.T.); 3 Polish Honey Chamber Association, 87-100 Toruń, Poland; przemyslaw@rujna.pl; 4 Institute of Management and Quality Sciences, Faculty of Economics, University of Warmia and Mazury in Olsztyn, 10-719 Olsztyn, Poland; 5 Department of Commodity Science and Food Analysis, Faculty of Food Sciences, University of Warmia and Mazury in Olsztyn, 10-719 Olsztyn, Poland; renap@uwm.edu.pl

**Keywords:** honey maturity, immature honey, osmophilic yeasts, water activity, fermentation risk, honey adulteration, metabolomic biomarkers, honey regulatory standards

## Abstract

Honey maturity is increasingly discussed in relation to product integrity, fair trade, and the classification of immature honey production as a form of adulteration. This narrative critical review examines honey maturity using evidence from peer-reviewed microbiological, physicochemical, and metabolomic studies, combined with an analysis of international and European regulatory frameworks, including Codex Alimentarius CXS 12-1981, Council Directive 2001/110/EC, Regulation (EC) No 178/2002, and Regulation (EC) No 852/2004. Particular attention is given to the interpretation of osmophilic yeast counts, water activity ($a_{w}$), moisture content, comb cell capping, fermentation, and technological dehumidification. The reviewed evidence indicates that osmophilic yeasts are natural components of honey and that their presence, expressed as colony-forming units per gram (CFU/g), should not be treated as an independent non-compliance criterion in the absence of active fermentation. Existing honey standards define compositional and quality requirements, including moisture, hydroxymethylfurfural, enzymatic activity, and absence of fermentation or effervescence, but do not establish a honey-specific CFU/g limit for yeasts. On this basis, the review formulates a functional maturity assessment framework integrating $a_{w}$, moisture, enzymatic indicators, and metabolomic biomarkers. The proposed framework is presented as a conceptual model derived from the synthesis of the existing literature and requiring further multilaboratory validation prior to adoption in official control practice. This approach may improve proportionality in honey quality assessment and reduce the risk of misclassifying microbiologically stable honeys as immature or adulterated.

## 1. Introduction

Honey is one of the oldest food products in human history, and at the same time one of the most frequently adulterated commodities on the modern food market. According to data from the European Food Fraud Database (FoodFraudNetwork), honey ranks third on the list of products with the highest adulteration risk, behind only milk and olive oil [1]. In 2023, the total volume of honey imports into the European Union exceeded 360,000 tonnes at a value close to EUR 1 billion [2], while the EU’s own production amounted to approximately 286,000 tonnes, covering only 60% of domestic demand [3]. The EU’s structural production deficit makes the market largely dependent on supplies from third countries, including China (35% of imports), Ukraine (31%), and Argentina (12%). The scale of this deficit, combined with the significant price disparity between authentic honey and syrup substitutes, creates economic incentives for fraudulent practices [1].

In the face of these challenges, Apimondia (the International Federation of Beekeepers’ Associations) plays a key role. This organization, uniting more than 80 national organizations and headquartered in Rome, constitutes a global forum for cooperation aimed at promoting scientific, technical, and economic progress in beekeeping [4].

The Apimondia position published in 2025 by the Working Group on Adulteration of Bee Products constitutes an important voice in the debate on product integrity [5]. The author identifies the systemic production of immature honey, defined as the deliberate collection of honey before the completion of the biological ripening process in the hive, combined with its industrial dehydration, as a separate and serious form of adulteration, distinct from classical practices of adulteration with sugar syrups [5]. The significance of this position is considerable: Apimondia, as the world’s largest federation of beekeeping organizations, has real influence on the shaping of both international standards and control practices applied by sanitary inspectorates in EU member states.

This article does not question the existence or seriousness of systemic immature honey production as a phenomenon violating the principles of fair food trade. The aim of this paper is, however, to indicate that the Apimondia position, perhaps inadvertently, makes an excessive generalization, equating three qualitatively distinct phenomena: (1) systemic, deliberate production of immature honey for economic gain; (2) early honey harvesting under conditions forced by climatic or technological factors; and (3) the presence of osmophilic yeasts as natural components of honey [5,6]. The consequence of this generalization is the creation of a disqualifying criterion based on the presence of yeasts, which remains in contradiction with both applicable regulatory standards and the current state of microbiological knowledge [7,8].

Analysis of applicable international standards, including Codex Alimentarius CODEX STAN 12-1981, EU Directive 2001/110/EC, and the draft ISO/DIS 24607:2025 [9] standard, leads to the conclusion that none of these documents establishes a limit on yeast cell count (CFU/g) as a criterion for assessing the quality or authenticity of honey [7]. The only legal microbiological criterion is fermentation as an active state, not merely the presence of microorganisms capable of fermentation [7].

Meanwhile, the fermentative activity of osmophilic yeasts, including *Zygosaccharomyces rouxii* and *Z. richteri*, is effectively inhibited at water activity $a_{w}<0.60$. In honey, this value is typically, but not invariably, associated with moisture content ≤ 20%, depending on sugar composition and other matrix-specific factors [10,11].

The above premises lead to the three-part thesis of this article: first, the presence of osmophilic yeasts in honey should be interpreted in relation to water activity and the absence of active fermentation; when $a_{w}<0.60$ is maintained, yeast growth and fermentation are effectively inhibited under standard storage conditions, whereas moisture content ≤ 20% should be treated as an important regulatory criterion but not as a strict surrogate for $a_{w}$; second, no applicable national or international standard penalizes the mere presence of yeasts without active fermentation, and therefore the determination of their count by the plate method (CFU/g) alone constitutes neither a legal nor a scientific basis for disqualifying the product from trade; third, the concept of functional maturity, understood as integrated microbiological, chemical, and biological stability, constitutes a more precise and operationalizable tool for honey quality assessment than the sole morphological criterion of comb cell capping [12,13,14].

The article is a review and covers the following areas:

1. Analysis of international regulatory frameworks concerning the microbiological parameters of honey;
2. Biology and metabolomics of the honey ripening process as a continuum;
3. Microbiology of osmophilic yeasts in the context of water activity and fermentation risk;
4. Diversity of global beekeeping production systems and their implications for standardization;
5. Analysis of the EU honey market and the regulatory consequences of overly strict interpretations;
6. A proposed framework for functional maturity assessment with recommendations for standardization bodies and Apimondia.

This article adopts a narrative critical review approach. It synthesizes peer-reviewed scientific literature and selected regulatory, standardization, and institutional documents relevant to honey maturity. Peer-reviewed literature was identified through searches of PubMed, Web of Science, and Scopus, using combinations of the following search terms: honey maturity, immature honey, osmophilic yeasts, water activity honey, honey fermentation, honey adulteration, honey metabolomics, honey ripening, and Zygosaccharomyces honey. The database search covered publications from 1980 to March 2026 and was not restricted by language at the query stage; however, the final synthesis included only English-language sources, as these could be reliably assessed by all authors. Regulatory and institutional documents were selected directly from official sources: FAO/WHO (Codex Alimentarius), the European Commission, ISO technical committees, national standards bodies, and major international beekeeping federations. A source was included if it directly addressed honey quality criteria, microbiological standards, or maturity assessment methodology. Particular attention is given to osmophilic yeasts, water activity, moisture content, fermentation risk, technological dehumidification, and honey adulteration. The reviewed sources include studies on honey microbiology, physicochemical and enzymatic indicators of ripening, metabolomic markers, international honey standards, and EU food law. The analysis is organized around four thematic areas: the regulatory interpretation of honey quality and fermentation, the biological and physicochemical mechanisms of honey ripening, the relationship between yeast counts and fermentation risk, and the applicability of maturity criteria under different climatic and production systems.

## 2. International Regulatory Frameworks: What Standards Actually Prohibit

The assessment of honey quality and authenticity is based on a network of interrelated normative documents of both global and regional scope. Understanding their scope and limitations is crucial for evaluating whether the presence of osmophilic yeasts can constitute an independent basis for disqualifying a product from commercial trade.

### 2.1. Codex Alimentarius, CODEX STAN 12-1981

The primary document regulating honey quality at the international level is the Codex Alimentarius Standard for Honey (CODEX STAN 12-1981, last revision 2023), developed jointly by FAO and WHO [7]. This standard defines honey as a natural product made by honey bees from the nectar of plants or from secretions of living parts of plants or excretions of plant-sucking insects on the living parts of plants, which the bees collect, transform by combining with specific substances of their own, deposit, dehydrate, store, and leave in the honeycomb to ripen and mature [7].

In terms of physicochemical parameters, CODEX STAN 12-1981 establishes the following quantitative limits: water content ≤ 20.0% (for heather honey *Calluna* ≤ 23.0%), content of hydroxymethylfurfural (HMF) ≤ 40 mg/kg (or ≤80 mg/kg for honeys from countries with a tropical climate), diastase activity ≥ 8 Schade units (or ≥3 Schade units for honeys with naturally low enzymatic activity, such as acacia honey *Robinia pseudoacacia*), and reducing sugar content [7]. The standard contains no limit on yeast count expressed in CFU/g. The only microbiological criterion is the prohibition on trading honey “that has begun to ferment or effervesce” [7]. This is a distinction of fundamental legal importance: the disqualifying criterion is the state of active fermentation, not the potential cause (the presence of microorganisms capable of fermentation).

### 2.2. EU Directive 2001/110/EC and National Law

At the European Union level, the primary legal act regulating honey trade is Council Directive 2001/110/EC of 20 December 2001 on honey, implemented in the Polish legal order by a regulation of the Minister of Agriculture and Rural Development [15]. This Directive, modeled on the Codex standard, does not establish a CFU/g limit for yeasts or fungi in honey. Microbiological parameters of honey are subject to general food hygiene provisions, in particular Regulation (EC) No. 852/2004 of the European Parliament and of the Council on the hygiene of foodstuffs [15,16].

In the control practice of veterinary and sanitary inspection laboratories, there is an unofficial hygiene threshold of 100 CFU/g for the total count of fungi and yeasts, used as a general indicator of production process hygiene; however, this threshold is not grounded in specific provisions regarding honey and cannot constitute an independent basis for declaring the product non-compliant with the requirements of food law within the meaning of Article 14 of Regulation (EC) No. 178/2002 [15]. Directive 2001/110/EC also permits the possibility of filtering, heating (to specified temperatures), and blending batches of honey as legal technological treatments, provided they do not lead to a change in its fundamental character [15].

### 2.3. United States Pharmacopeia (USP) and Other National Standards

The United States Pharmacopeia (USP) classifies honey as an excipient for pharmaceutical and food use. USP requirements include physicochemical parameters similar to those of Codex but also do not contain a CFU/g limit for osmophilic yeasts [17]. A similar approach is taken by national standards applicable in the major honey-exporting countries—China (GH/T 18796-2012) [18], Argentina (Código Alimentario Argentino, CAA), and Brazil (IN MAPA No. 89/2000) [19]—which define honey quality by chemical parameters and enzymatic activity, not by yeast count [17].

### 2.4. Comparative Overview of Standards

Table 1 presents a comparison of key honey quality parameters in applicable international and regional standards with respect to microbiological criteria.

### 2.5. Regulatory Conclusion

Analysis of the above documents leads to an unequivocal conclusion: no applicable national or international standard treats the presence of osmophilic yeasts per se as a basis for disqualifying honey from commercial trade. The disqualifying criterion is exclusively the biological outcome (active fermentation and effervescence), not the potential cause (the presence of microorganisms capable of fermentation).

This distinction is of fundamental practical importance: honey in which osmophilic yeasts are present at any measured count should not be disqualified on this basis alone, provided that water activity $a_{w}<0.60$ is maintained, no signs of active fermentation are observed, and other compositional criteria, including the applicable moisture limit, are met [7,15].

A CFU/g measurement alone, without a simultaneous assessment of water activity ($a_{w}$), temperature, and storage time, is methodologically insufficient for declaring the product non-compliant with the requirements of food law [17,20].

## 3. Biology of Honey Ripening: A Continuous Process, Not a Binary Event

Honey ripening is a complex biotransformation process in which nectar or honeydew undergoes a series of enzymatic, physicochemical, and microbiological transformations before reaching a state of qualitative stability. The widespread equating of honey maturity with the fact of comb cell capping with wax—common in beekeeping practice and veterinary control—constitutes a simplification that is inadequate from a biochemical perspective. Capping is one of the final, morphological indicators of ripening, not synonymous with the achievement of full chemical and microbiological stability [12,14].

### 3.1. Enzymatic Transformation of Nectar

The honey production process is initiated at the very moment nectar or honeydew is collected by forager bees.

Nectar, dominated by sucrose and containing a high water content, undergoes the first enzymatic transformation in the honey crop of the insect, where it mixes with secretions from the bee’s hypopharyngeal glands [21]. The key enzymes initiating the transformation are:

- Invertase (β-D-fructosidase): It catalyzes the hydrolysis of sucrose into fructose and glucose; produced by the hypopharyngeal glands of worker bees [21]; its activity increases gradually from low values in immature honey to maximum values in mature honey, making it one of the most sensitive indicators of the degree of maturity [12].
- Glucose oxidase: It oxidizes glucose to gluconic acid with the release of hydrogen peroxide (H_2_O_2_); gluconic acid is responsible for lowering honey pH to the range of 3.5–4.5 and constitutes the dominant organic acid in mature honey; H_2_O_2_ serves as a natural antibacterial agent [12,21].
- Diastase (α- and β-amylase): It catalyzes the hydrolysis of starch and nectar polysaccharides into glucose and maltose; diastase activity is a parameter standardized by Codex Alimentarius (≥8 Schade units) and serves as an indirect indicator of thermal integrity and honey maturity [7,12,21].
- Catalase: It decomposes excess H_2_O_2_, regulating the balance between antibacterial activity and protection of the remaining honey components from oxidation [21].

Studies of enzymatic activity at five successive stages of transformation—from floral nectar, through the honey crop of foragers and house bees, to uncapped and capped hive cells—showed a gradual increase in invertase, amylase, and glucose oxidase activity at each successive stage of processing [22]. These data confirm that enzymatic transformation proceeds as a continuum along the entire production chain, not as a step-change event occurring at the moment of cell capping [12,22].

In parallel with enzymatic transformations, house bees actively dehydrate honey through hive ventilation: wing beats create a constant airflow over open comb cells, accelerating water evaporation and reducing the moisture of the raw material from values characteristic of nectar to a final approximately 18.5% in honey before capping [12]. Full maturation under natural conditions typically takes several days to a few weeks, with this time potentially being significantly extended under conditions of high relative humidity [12,23].

### 3.2. Ripening as a Process: Metabolomic Evidence

The thesis of the binary nature of honey maturity—according to which honey is either immature (uncapped) or mature (capped)—finds increasingly weak support in current metabolomic research. Sun et al. [14] in a study of rapeseed honey (*Brassica napus*) demonstrated that maturity indicators, including water content, fructose-to-glucose ratio, invertase and diastase activity, and organic acid content, change gradually during the ripening process and reach stable values independently of the moment of cell capping by bees.

Guo et al. [13] conducted a comprehensive comparative analysis of the chemical profile and biological activity of mature and immature honeys using HPLC/QTOF/MS-based metabolomics. The authors identified significant differences in polyphenol content, antioxidant activity (DPPH, FRAP), and amino acid profile between honey collected in an immature state and mature honey; however, these differences were closely correlated with chemical parameters (water content and enzymatic activity) rather than solely with the fact of capping [13]. Importantly, some samples of honey collected before full capping exhibited a metabolomic profile similar to mature honey, provided they met the criterion of low moisture, which constitutes direct confirmation of the concept of functional maturity.

Sun et al. [14] using GC-MS and LC-MS identified decenedioic acid, a fatty acid of bee origin, as a potential molecular biomarker differentiating mature from immature honey. This biomarker, validated for rapeseed, acacia, and jujube honeys, was significantly more abundant in mature honey (p<0.01), confirming that ripening is a process of gradual accumulation of specific metabolites, not a binary transition [14].

Beyond fatty acid biomarkers, the metabolomic fingerprint of honey maturity encompasses several additional compound classes. Polyamines, particularly spermidine and spermine of plant and bee origin, accumulate progressively during ripening and have been proposed as markers of enzymatic activity and cellular integrity in the maturing product [13,21]. Flavonoids (quercetin, kaempferol, and luteolin) and phenolic glycosides derived from floral nectar undergo concentration and partial transformation during the ripening process; their profiles are correlated with diastase and invertase activity and constitute part of the antioxidant capacity indexed by DPPH and FRAP assays [13]. Organic acids, including gluconic, citric, malic, and succinic acids, increase in concentration as glucose oxidase activity rises during ripening, providing both a pH indicator and a chemical maturity marker independent of moisture content [12,21]. These compound classes have been identified in multiple botanical types and are analytically accessible by LC-MS and NMR-based metabolomics platforms [24], supporting their potential inclusion in a multiparametric maturity assessment framework.

It is worth emphasizing that exposure of honey to high temperatures, even after full capping, can cause enzyme degradation and effectively revert quality parameters to values characteristic of immature honey [21]. Capped honey subjected to inappropriate heat treatment may exhibit diastase activity below the Codex threshold (<8 Schade units) and elevated HMF content, demonstrating that the morphological criterion of capping does not guarantee the maintenance of functional maturity throughout the supply chain.

This is also confirmed by beekeeping practice, which treats the fact of cell capping merely as an indicator of potential honey maturity. On one hand, it sometimes happens that under certain conditions bees cap comb cells in which honey is not yet mature (water content exceeds 20% and shortly after extraction the honey ferments). On the other hand, common practice involves harvesting honey from partially capped frames (usually with a threshold of three-quarters of cells capped in the frame), provided that in the uncapped cells the honey is mature. The actual indicator of honey maturity is the water content, which the beekeeper determines either by refractometry or by other tests (e.g., shaking the frame), whose result is directly dependent on water content.

### 3.3. The Concept of Functional Maturity

Based on the above review, functional honey maturity can be operationally defined as an integrated product state characterized by the simultaneous fulfillment of three groups of criteria:

1. Microbiological stability: absence of active fermentation; water activity $a_{w}<0.60$; moisture ≤ 20%; osmophilic yeast count below the growth threshold under given conditions of temperature and storage [10].
2. Chemical stability: fructose-to-glucose ratio (F/G) ≥ 0.9; HMF content ≤ 40 mg/kg; diastase activity ≥ 8 Schade units; invertase activity ≥ 4 units; gluconic acid content and organic acid profile within the reference range for the given botanical type [7,12].
3. Biological stability: antioxidant activity (DPPH, FRAP) at the level appropriate for the given botanical and geographical type; polyphenol and flavonoid content not reduced relative to reference values; antibacterial activity (assessed by H_2_O_2_ production and the presence of defensins) unchanged [13].

This definition deliberately separates the morphological criterion (cell capping) from the quality criterion, recognizing that honey may achieve functional maturity before full comb capping (e.g., at 18% moisture in a temperate climate). This concept is consistent with the Codex regulatory approach, which, as demonstrated in Section 2, penalizes the state of fermentation, not morphology [7,14].

## 4. Microbiology of Yeasts in Honey: Presence vs. Activity vs. Adulteration

The microbiology of honey is a field relatively rarely analyzed in the context of product authenticity assessment, despite the fact that microorganisms—among them osmophilic yeasts—are an inseparable component of every honey produced under natural conditions. This section states and justifies the central microbiological thesis of the article: the mere presence of osmophilic yeasts in honey is a biologically normal phenomenon, and does not constitute grounds for declaring the product immature, let alone adulterated, provided that water activity ($a_{w}$) and water content are maintained at levels prescribed by applicable standards.

### 4.1. Osmophilic Yeasts as a Natural Component of Honey

#### 4.1.1. Biological Characteristics of Osmophilic Yeasts

Osmophilic yeasts, also called osmotolerant or xerotolerant, are microorganisms capable of growth and metabolism in environments with very high osmotic pressure, i.e., at water activity $a_{w}$ close to 0.60–0.62 [10,11]. In honey, the dominant role is played primarily by species of the genus *Zygosaccharomyces*: *Zygosaccharomyces rouxii* (Boutroux) Yarrow, first described in 1893 as yeasts isolated from sucrose solutions, constitutes the model species for fungal osmotolerance. Its minimum water activity for growth is $a_{w}=0.62$, making it one of the most resistant osmotolerant fungi known to science [10]. Under conditions below this threshold, *Z. rouxii* remains in a state of cryptobiosis; cells retain viability and germination capacity but show no metabolic activity or growth [10,11].

More recent studies using culture-dependent and culture-independent methods (metagenomics) have expanded the list of yeasts isolated from honey to include further unconventional species, including *Candida* spp., *Rhodosporidiobolus ruineniae*, *Clavispora lusitaniae*, and *Metschnikowia chrysoperlae* [8]. Metagenomic studies indicate that *Zygosaccharomyces* remains the dominant genus in both western honey bee (*Apis mellifera*) honeys and eastern honey bee (*Apis cerana*) honeys, regardless of country of origin [25].

#### 4.1.2. Sources of Colonization and Prevalence

Osmophilic yeasts are an integral component of the hive ecosystem and beekeeping environment, which not only precludes the justifiability of imposing restrictions in this regard but also excludes the possibility of their elimination from honey without applying extreme technological interventions. The main colonization routes include floral nectar, in which yeast counts are typically $10^{2}$–$10^{4}$ CFU/ml depending on the plant species, microclimate, and season; pollen and bee bread, which are carriers of active yeast and lactic acid bacteria populations; as well as the bee’s digestive tract—the honey crop and intestine constitute a natural reservoir of microorganisms [26]. Additional sources include soil, wax, and hive surfaces, whose yeast biofilm is difficult to eliminate without disinfectants incompatible with organic production [26].

The presence of osmophilic yeasts in honey is therefore an inevitable phenomenon resulting from the biology of the production process, not from production deficiencies. This is confirmed by the literature from various climatic and geographical zones, encompassing both western and eastern honey bee honeys [25]. The volume of yeasts found in normal non-fermenting commercial honey typically ranges from several dozen to several hundred CFU/g; values above 1000 CFU/g are recorded in correct samples without signs of fermentation, provided moisture does not exceed 18% [27]. To provide concrete empirical context, Ruegg and Blanc [11] reported osmophilic yeast counts of 10–$10^{3}$ CFU/g in commercially traded European honeys showing no fermentation signs, and Rodriguez-Machado et al. [8] identified counts ranging from below detection limits to $1.5\times 10^{3}$ CFU/g across 120 honey samples of diverse botanical origin, none of which showed active fermentation at moisture ≤ 18%. Metagenomic surveys confirm that *Zygosaccharomyces* populations in this CFU/g range represent a stable, metabolically inactive reservoir under $a_{w}<0.60$ conditions [25].

### 4.2. Water Activity as a Critical Control Parameter

#### 4.2.1. Definition and Significance of Water Activity in Honey

Water activity ($a_{w}$) is a thermodynamic measure of the availability of water in a food product for chemical reactions and microbiological activity, defined as the ratio of the vapor pressure above the product to the vapor pressure of pure water at saturation at the same temperature [10]. In contrast to water content (moisture), expressed as mass percentage, $a_{w}$ reflects the actual availability of water to microorganisms and is the primary parameter determining microbial growth, enzymatic activity, and non-enzymatic browning of the product [10].

Honey is a supersaturated solution of simple sugars, fructose (∼38%) and glucose (∼30%), and higher oligosaccharides, in an environment of minimal water content. The high osmolarity of the solution resulting from this saturation causes water molecules to be strongly bound to sugar molecules, and their thermodynamic activity is drastically reduced [10]. In honey with moisture ≤ 20%, water activity is typically $a_{w}<0.60$, although the exact value depends on sugar composition, water content, and measurement temperature [11,28]. The study of Ruegg and Blanc [11], cited by all subsequent food microbiology textbooks as a fundamental reference for honey microbiology, demonstrated that for the moisture range of 16–21% water activity falls between $a_{w}=0.56$ and $a_{w}=0.63$, with the value $a_{w}=0.60$ corresponding to moisture of ≈17.5–18.5% depending on sugar composition.

#### 4.2.2. Osmophilic Yeast Growth Threshold and Water Activity

The minimum water activity required for osmophilic yeast growth is $a_{w}=0.61$–0.62. Below this threshold, yeast growth is effectively inhibited under standard storage conditions, and cells may remain viable but metabolically inactive or markedly restricted in activity [11,26]. In the context of honey, this means that in products with $a_{w}<0.60$, osmophilic yeasts are not expected to grow or initiate fermentation under normal commercial storage conditions, irrespective of the measured CFU/g count.

The practical implication is direct: honey containing even $10^{4}$ CFU/g of osmophilic yeasts but with moisture of approximately 17% and ($a_{w}\approx 0.57$–0.58) should remain microbiologically stable under standard storage conditions [26].

The Analytica Laboratories document formulates this principle as a multiparametric model: fermentation is highly probable only at moisture > 19% and yeast count > 10 CFU/g simultaneously [27], which a contrario precludes product disqualification on the basis of the CFU/g parameter alone.

For reasons of microbiological stability, water activity is an incomparably more important parameter than the percentage water content. It should be noted that for certain types of honey (e.g., heather honey), legal requirements permit water content levels above 20%. This is due to the fact that the specific composition of these honeys ensures microbiological stability at water content exceeding 20%, and these honeys should simultaneously be considered “mature” at the values specified in legal requirements. If water activity studies demonstrate that the water activity of these honeys does not exceed the threshold ($a_{w}<0.60$), this parameter would constitute a significantly more universal parameter for determining the microbiological stability and degree of maturity of honey.

#### 4.2.3. Glucose Crystallization as a Dynamic Factor Modifying Water Activity

The natural process of glucose crystallization in honey is a factor deserving particular attention in the context of fermentation risk, as it constitutes a mechanism by which initially microbiologically stable honey can become susceptible to fermentation without any external intervention. During crystallization, glucose transitions from the liquid phase to the solid phase as glucose monohydrate (C_6_H_12_O_6_ · H_2_O). Each glucose molecule binds one water molecule, effectively removing it from the liquid phase [10]. However, the liquid phase remaining after glucose crystallization becomes enriched in fructose and water, causing an increase in water activity in the remaining liquid phase, even though the total water content in the sample does not change [10,29].

Zamora and Chirife [10] in a study of 50 samples of crystallized and re-dissolved Argentine honeys demonstrated that crystallization causes an increase in the $a_{w}$ of the liquid phase by 0.03–0.04 units compared to honey in the liquid state. For honeys with initial moisture of 17.5–18.0% ($a_{w}\approx 0.58$–0.59), crystallization can shift the $a_{w}$ of the liquid phase above the threshold of 0.62, thereby activating the osmophilic yeast population present in the product [10,29]. This phenomenon leads to a key methodological conclusion: the microbiological control of honey must take into account the phase state of the product (liquid vs. crystallized) since the same CFU/g count carries different fermentation risk depending on whether the honey is liquid or crystallized. These processes further support the need to interpret yeast counts together with water activity and the physical state of honey.

Given that honey crystallization proceeds in a differentiated manner (i.e., through different crystal forms with regard to both structure and dynamics of formation), changes in water activity and thus the potential for yeast proliferation in crystallizing honey are variable. This variability is observed not only with respect to the time/progress of crystallization but also the sampling point within the container. Considering that crystallization propagation occurs at the molecular level, the greatest potential for water concentration occurs in the pericrystalline and intercrystalline spaces. Mainly through diffusion, this fluctuation tends to equalize in areas increasingly distant from the crystal surface. It should be noted that the interphase equilibrium is a dynamic process in which the direction of phase transitions is determined by temperature (variable storage conditions). Depending on the conditions accompanying these phase transitions (such as temperature, solution density, oxygen availability, honey aeration, the free spaces formed in honey during crystallization, the rate of crystallization resulting from the proportions of individual sugars, etc.), the potential for yeast proliferation in solution is dynamic and varies throughout the volume of honey. A CFU/g measurement without precise specification of the phase state and $a_{w}$ is therefore methodologically incomplete.

From the above it follows that if water activity could determine honey maturity, if this parameter were specified in legal requirements, the water activity value should be set at a level achievable at the time of honey extraction, and should also be maintained (without further technical measures) despite differences in temperature and phase transitions occurring during storage and distribution.

### 4.3. Yeast Count as a Risk Indicator, Not a Disqualifying Parameter

#### 4.3.1. Multiparametric Model of Fermentation Risk

Analysis of the scientific literature and practical documents indicates the widespread acceptance of a multiparametric model of honey fermentation risk, in which no single indicator is sufficient to determine the microbiological stability or instability of the product. Fermentation risk is a function of at least four variables:

(1) $$R_{f}=f\left(a_{w},\;T,\;t,\;N_{CFU}\right)$$

where $R_{f}$ is fermentation risk (probability of initiating active fermentation), $a_{w}$ is water activity, *T* is storage temperature [°C], *t* is storage time [days], and $N_{CFU}$ is the osmophilic yeast count [CFU/g].

According to this model, yeasts can ferment honey only when all growth-promoting parameters are simultaneously fulfilled: $a_{w}$ must exceed the threshold of 0.62, temperature must fall within the optimal range for yeasts (10–27 °C), and the exposure time must be sufficient to reach the cell density initiating noticeable fermentation [30]. The New Zealand Beekeeping Industry document states directly that honey with moisture > 19% and yeast count > 10 CFU/g is susceptible to fermentation, which a contrario implies that honey with moisture < 17%, even with a substantially higher yeast count, is not expected to ferment under normal commercial storage conditions [30].

Table 2 illustrates the matrix model of fermentation risk as a function of moisture and yeast count, based on available empirical data.

The risk categories presented in Table 2 are heuristic estimates compiled from two practitioner-oriented documents [27,30] and represent the consensus of applied honey quality practice. They have not been derived from a controlled experimental study under defined storage conditions; the term “no risk” should be understood as indicating negligible probability of fermentation initiation under normal commercial storage, not an absolute thermodynamic guarantee. The table is intended as a decision-support tool and should be interpreted alongside direct $a_{w}$ measurement rather than used as a standalone classification system.

#### 4.3.2. Limitations of the Plate Method (CFU/g) as a Risk Assessment Tool

The standard enumeration of yeasts and molds in foods is commonly based on plating serial dilutions on selective media, followed by incubation and colony counting.

This method, while widely used and well-validated, has a number of significant limitations in the context of honey fermentation risk assessment:

1. Failure to distinguish viable from dead cells: the plate method counts only cells capable of forming colonies on solid medium under incubation conditions; living cells in a state of deep anabiosis induced by low honey $a_{w}$ may fail to grow on the medium or grow atypically, leading to underestimation of the true yeast count;
2. Lack of correlation with in situ $a_{w}$: the CFU/g result is a measure of the potential population size but does not indicate whether under the conditions prevailing in the tested product this population is active; only the combination of CFU/g with $a_{w}$ measurement allows assessment of the actual fermentation risk [11];
3. Lack of species specificity: standard selective media do not differentiate yeast species with different fermentation potential; *Metschnikowia* spp. with low fermentation potential are counted identically to *Z. rouxii* with high potential [8].
4. Legal–scientific conclusion: in light of the above methodological limitations, the mere determination of yeast count (CFU/g) in honey, without a simultaneous measurement of $a_{w}$, determination of the product’s phase state, storage temperature, and time since harvest, does not constitute a sufficient scientific basis for assessing fermentation risk, let alone for disqualifying the product from commercial trade. Given that no applicable standard (Codex, EU, ISO) establishes a CFU/g limit for honey (cf. Section 2), a potential inspector’s decision to disqualify a product based solely on CFU/g count would be devoid of both scientific and legal grounds.

### 4.4. Fermentation as a Dynamic and Post-Harvest Event

The Apimondia position and much of the literature on honey adulteration tacitly assume that fermentation detected in honey is evidence of its immaturity at the time of harvest. This assumption is, however, incorrect from the perspective of microbiological dynamics since fermentation is an event that can occur at any stage of the supply chain, not only at the time the product leaves the hive [27,30].

Correctly harvested honey that meets all normative criteria, with moisture of, for example, 18.5% ($a_{w}\approx 0.60$) and a yeast count of 200 CFU/g, stored for six months in a warehouse at a temperature of 22 °C and relative humidity above 70%, may locally exhibit higher water activity (crystallization, temperature changes, air access, etc.), shifting the $a_{w}$ above the yeast growth threshold and initiating fermentation [30].

Border inspection, sampling imported honey after several months of transport and storage, takes measurements at a point removed in time and space from the moment of harvest; the determination of fermentation or elevated active yeast count at that moment does not allow, without analysis of temperature and humidity documentation in the supply chain (cold chain monitoring), reliable reconstruction of the product’s state at the time of honey extraction (verifying the degree of capping) or departure from the apiary [17,20]. Therefore, fermentation observed at the point of inspection should not be treated, by itself, as proof of deliberate adulteration at the production stage. It may indicate product instability or non-compliance, but attribution to intentional immature honey production requires additional evidence from the extraction, processing, storage, or supply-chain stages.

Deliberate adulteration consisting of harvesting immature honey followed by technological treatments aimed at its microbiological stabilization can only be established through controls at the honey extraction stage, and effective limitation may be achieved by specifying prohibited technologies (e.g., vacuum dehydration). The non-use of prohibited technologies should be a mandatory stage of official controls of apiaries and establishments involved in honey extraction and placing on the market. Neither yeast count nor fermentation permits inference about this.

## 5. Global Production Systems and Climatic Conditions

Honey quality standards, including the Apimondia position, were developed on the basis of the European temperate climate and frame beekeeping, yet more than 70% of global honey production comes from regions that are climatically, technologically, and culturally different from Europe [5,15]. Applying uniform morphological and microbiological criteria to honeys from different production systems is methodologically limited and may create disproportionate assessment outcomes for producers operating under different climatic and technological conditions [23,31].

### 5.1. Global Landscape of Apicultural Production

According to FAO data from 2024, the global number of managed colonies exceeded 101.7 million, with Asia dominating at a 44.4% share, and Europe holding 24.7% [32]. Honey production in 2022 reached 1.83 million tonnes, with Asia accounting for 48.2%, Europe for 22.8%, the Americas for 18.5%, and Africa for 8.5% [31]. Adopting the capping criterion as the sole determinant of maturity in practice favors European honeys and discriminates against honeys from tropical and traditional systems [23,31].

Relative humidity in the tropical zone of Africa is typically 70–90% RH, exceeding 95% RH during the rainy season. Under such conditions, bees are not biologically capable of reducing honey moisture to ≤20% solely through natural hive ventilation [23]. The beekeeper faces a choice: early harvesting and technological dehumidification, or the risk of in situ fermentation or plundering by pests. Tadele et al. [23] classify dehumidification as a technological necessity, not an intent to adulterate, emphasizing that the alternative is crop loss or fermented product. Studies show that honey subjected to proper dehumidification retains full enzymatic and antioxidant profile [33,34].

#### African Production: Climatic Humidity and Traditional Hive Systems

In Ethiopia, 95.5% of hives are traditional, with only 0.2% being frame hives [35]. Paradoxically, the study by Gobessa et al. [36] demonstrated that honey from traditional hives is characterized by significantly lower moisture than from modern hives (p<0.001), as harvesting occurs once or twice per year, giving more time for natural evaporation. At the same time, traditional hives make frame inspection and assessment of the degree of capping impossible without destroying the combs; applying a frame-based morphological criterion to these systems illustrates the limited transferability of maturity indicators derived mainly from temperate-climate European production systems [23].

### 5.2. Honeys of Stingless Tropical Bees

Stingless bees (Meliponini, >500 species) produce honey with moisture typically of 25–35% and $a_{w}=0.60$–0.86, a different sugar profile, and higher acidity than *Apis mellifera* honeys [37,38]. Meliponini do not possess nectar dehydration mechanisms comparable to the European bee: smaller honey crops and less developed hive ventilation are biological characteristics, not production deficiencies. A study of 17 species from Brazil [37] demonstrated a range of $a_{w}=0.60$–0.86, with osmophilic yeasts isolated from these honeys characterized by high osmotolerance and low fermentative capacity—biological adaptation to an environment with elevated $a_{w}$. Meliponini honeys require separate standardization; assessing their quality on the basis of *Apis mellifera* standards is biologically unjustified [37,38].

### 5.3. Specific Microclimates and Weather Conditions in the Temperate Zone

Honey extraction with moisture levels that pose a real risk of fermentation also occurs in the temperate zone, including in Europe. Many areas from which honey is harvested are characterized by a specific microclimate with persistently elevated air humidity. Weather factors such as rainfall lasting for periods of several days to several weeks influence elevated water content in combs. This is precisely why the Apimondia position states that a justified beekeeping practice is to leave honey after extraction in open vessels for the purpose of evaporating excess water. The extraction of honey with elevated water content is therefore a fact, as is human intervention aimed at removing excess water for the purpose of microbiological stabilization. Commonly practiced by European beekeepers is not only the periodic mixing of honey to increase the rate of evaporation but also the use of various technical devices for this purpose (e.g., rotating discs that increase the evaporation surface). Technical development and process automation are visible at all stages of honey extraction and serve not only to increase efficiency but also effectively enhance product safety. Unquestionably, however, they cannot be instruments of honey adulteration. Therefore, clear, accessible, and verified criteria contained in normative acts should underpin the assessment of such activities, and within justified scope, prohibited practices should be precisely specified (e.g., the use of nanofiltration, ion exchange resins, and vacuum dehydration).

### 5.4. Comparison of Production Systems

Table 3 summarizes the key characteristics of the three globally dominant hive types. Only the Langstroth hive provides full control over honey ripening; KTBH and traditional hive systems are structurally limited in this respect, meaning that honeys from these systems may fail to meet the capping criterion while complying with all good beekeeping practices [23,36,39].

### 5.5. Limitations of the Morphological Capping Criterion

Based on the above analysis, the criterion of full capping as the sole determinant of honey maturity has limited global applicability and may be technically inadequate in several production systems for five reasons: (1) the climatic impossibility of fully dehydrating nectar under conditions of 70–90% RH humidity [23]; (2) the technological inaccessibility of capping assessment in traditional hives without comb destruction [36]; (3) the biological distinctness of Meliponini honeys that precludes direct application of *Apis* standards [37]; (4) the empirical inconsistency between the degree of capping and the microbiological stability of honey [12,13]; and (5) the impossibility of gathering, archiving, and verifying evidence, and of post factum proof from the degree of capping. It is necessary to adopt an integrated model for assessing functional maturity based on measurable parameters ($a_{w}$), not on a morphological criterion [7,11,23].

## 6. EU Honey Market and Regulatory Implications

The European honey market is the world’s largest regional import market, and its structural characteristics—chronic domestic production deficit, high dependence on imports from third countries, and wide quality variation of supplied products—constitute a context in which regulatory decisions concerning honey disqualification criteria have direct economic, trade, and consumer consequences. This section analyzes this market context as justification for the precise approach advocated in this article, based on functional maturity and water activity, in place of an overly simplified approach based on a yeast count or morphological criterion.

### 6.1. Structure and Dynamics of Honey Imports into the EU

The European Union is the world’s largest regional honey importer, generating a trade balance deficit in the beekeeping sector that has no equivalent in any other region [2,3]. EU production in the 27 Member States was estimated at approximately 272,286 tonnes in 2022–2023, covering only 55–60% of domestic demand, estimated at approximately 480–500 thousand tonnes [3,40]. The difference is filled by imports from third countries.

In 2023, extra-EU honey imports amounted to approximately 163,700 tonnes, while the broader volume of honey entering and circulating within the EU supply chain, including intra-EU trade, was estimated at approximately 300,000–360,000 tonnes [2].

In 2024, total imports (extra-EU + intra-Community) amounted to approximately 290 thousand tonnes (a decline of 3.1% year-on-year after three years of growth), but the long-term trend remains upward: the EU market is expected to reach a volume of 445 thousand tonnes by 2035 (CAGR +1.9%) [41].

The main external suppliers in 2023 were: China (37% of extra-EU imports), Ukraine (28%), Argentina (approx. 7%), Mexico and Cuba [42]. The largest national importers within the EU were Germany (41 thousand tonnes, 25% of extra-EU imports), Belgium (31.4 thousand tonnes, 19%), Poland (23.3 thousand tonnes, 14%), Spain (15.7 thousand tonnes), and France (7.7 thousand tonnes) [41].

### 6.2. The Scale of Honey Adulteration on the EU Market: Empirical Data

The scale of the honey authenticity problem on the EU market is documented by a number of studies conducted at the institutional level. The coordinated control action of the European Commission “From the Hives” (2021–2022), covering analysis of 320 samples of imported honey, demonstrated that 46% of the analyzed samples were suspected of non-compliance with the Honey Directive, representing a threefold increase compared to a similar study from 2015 to 2017, when the figure was 14% [43]. The highest absolute percentage of suspect shipments concerned China (74%), while Turkey showed the highest relative rate (93%) [43]. Among the identified adulteration practices, the addition of sugar syrups (from sugarcane, corn, and rice) dominated, along with blending authentic honey with syrup products in proportions below the detection threshold of conventional methods [20,43].

These data indicate a real and serious honey authenticity problem on the EU market but simultaneously clearly profile the dominant form of adulteration as sugar syrup adulteration, not as the distribution of honey with an elevated yeast count. JRC and OLAF documents do not indicate a single case of honey disqualification based solely on the yeast CFU/g count without simultaneous determination of active fermentation or syrup adulteration [43]. This is a significant observation from the perspective of this article: adulteration practices focus on the chemical dilution of honey, not on the manipulation of its microflora.

In response to this crisis, the European Commission adopted Directive (EU) 2024/1438 (part of the “Breakfast Directives” package), introducing from 2026 an obligation to declare all countries of origin of honey blend components in the principal field of view of the label [44]. The implementation of this requirement strengthens the traceability infrastructure but does not resolve the problem of the lack of validated parameters specific to immaturity.

### 6.3. Analytical Gaps: Absence of a Validated Immaturity Biomarker

The identification of immature honey as a distinct category of adulteration requires the existence of analytical tools capable of reliably distinguishing between immature and mature honey, and here science encounters a fundamental limitation. As the review by Tsagkaris et al. [17] demonstrated, standard physicochemical methods (moisture, diastase, invertase, HMF, and sugar profile) are not sufficient for unambiguous determination of immaturity, because:

1. There is no specific marker: no single chemical compound or enzyme is identified as a biomarker exclusively of immaturity in a manner independent of botanical and geographical type; the physicochemical parameters of mature honey from the tropics may resemble the parameters of immature honey from the temperate zone [13,17].
2. Metagenomics and metabolomics have limited discriminatory power without reference databases: as Schoder [20] emphasizes in his 2026 review, the high analytical resolution of omic methods (genomics, proteomics, NMR, GC-MS, and LC-HRMS) does not guarantee unambiguous fraud attribution without solid, globally representative reference databases; “high analytical resolution alone therefore does not guarantee unambiguous fraud attribution” [20].
3. Blending effects mask immaturity: blending immature with mature honeys in proportions below 50% is analytically indistinguishable from mature honey by conventional methods [20]; it should be emphasized that this problem also applies to the honey extraction stage—it is impossible to determine what fraction of honey was mature and what was immature at the time of extraction.
4. Yeast presence is not a specific marker: as demonstrated in Section 4, the osmophilic yeast count in honey is determined mainly by climatic production conditions and hive systems, not by the degree of maturity, which excludes CFU/g as a useful immaturity biomarker [8,20].

The most recent studies point to promising directions in the search for immaturity biomarkers: Sun et al. [14] identified decenedioic acid and other fatty acids of bee origin as potential molecular markers whose concentration correlates with the degree of maturity in a manner independent of yeast count. NMR-based metabolomics [24] enables simultaneous quantification of several dozen compounds in a single measurement, providing a basis for developing a multidimensional maturity index. However, to date, none of the proposed biomarkers has undergone full multilaboratory validation encompassing the global diversity of botanical and geographical types [17].

It should be borne in mind that legally established methods should be as simple, rapid, and accessible as possible since they will constitute the basic tool of every beekeeper (similar to the current refractometer) for determining the timing of extraction, as it is this stage that determines whether honey is mature or not.

### 6.4. Regulatory Risk: Consequences of Overly Strict Interpretation

Adopting a disqualification criterion based on yeast CFU/g count, without taking into account water activity, climatic production conditions, and the absence of active fermentation entails a number of serious regulatory and market risks, whose analysis is necessary for a proper assessment of the implications of the Apimondia position:

- Risk of discrimination against tropical producers.

As demonstrated in Section 5, honeys from tropical and traditional systems naturally contain higher concentrations of osmophilic yeasts due to hive type, climatic conditions, and richer endemic microbiological biodiversity [23,37]. Disqualification of these honeys based on CFU/g would effectively result in excluding from the EU market products legally produced in countries of East Africa, Southeast Asia, and Latin America, while not affecting syrup-adulterated honeys (which contain a standard or reduced yeast count following the ultrafiltration process) [43].

- Risk of aggravating the deficit and price increases.

Excluding from the EU market honeys from China, Ukraine, and Argentina—the three largest suppliers collectively accounting for ≈72% of extra-EU imports [42]—based on the CFU/g criterion would cause a sharp rise in honey prices on the European market and an aggravation of the deficit, particularly severe for consumers in countries with low domestic consumption (Poland, Germany, and Belgium as net importers) [41].

- Risk of creating arbitrary trade barriers.

Applying a criterion that has no basis in Codex Alimentarius or EU Directive 2001/110/EC can be challenged by trading partners under the WTO-SPS Agreement (Agreement on the Application of Sanitary and Phytosanitary Measures), which requires that trade-restrictive measures have a scientific basis and be proportionate to the identified risk [7,15]. The risk of harvesting immature honey exists for all honeys, regardless of where the apiary is located. Introduced requirements and restrictions should have a global dimension and their proportional reflection in EU law with respect to EU honey producers.

- Risk of missing the actual problem.

As demonstrated by the “From the Hives” action, the dominant form of honey adulteration in the EU is sugar syrup adulteration, not the distribution of immature honey with an elevated yeast count [43]. Focusing inspection resources on CFU/g measurement at the expense of isotope ratio analysis ($\delta^{13}$C, $\delta^{2}$H, $\delta^{18}$O) and NMR fingerprinting would lead to the allocation of inspection resources inconsistent with the actual adulteration risk profile. It should be noted, however, that the European Commission later withdrew from treating ^1^H-NMR profiling as a sufficiently established official control method, emphasizing the need for further research, validation, and harmonization of analytical methods [45].

### 6.5. Regulatory Proposals: Harmonization, Traceability, and Proportionality

Based on the above market and regulatory analysis, this article formulates the following proposals addressed to standardization bodies (ISO, Codex), EU institutions (DG SANTE, EC), and industry organizations (Apimondia, Copa-Cogeca):

1. Update EU Directive 2001/110/EC with a water activity ($a_{w}$) criterion: introduction of mandatory $a_{w}$ measurement alongside moisture content as a primary parameter for fermentation risk assessment. Measurement of $a_{w}$ is a validated, commercially available method (instruments such as AquaLab, Rotronic HygroLab) and inexpensive; this would constitute a substitution of the morphological criterion with a physicochemical criterion having direct scientific justification [10,11].
2. Establish a multiparametric microbiological assessment protocol: fermentation risk assessment should be conducted simultaneously for $a_{w}$, water content, storage temperature, and yeast count (in accordance with the risk Equation (1) proposed in Section 4.3); none of these parameters may be decisive in isolation from the others [27,30].

## 7. Proposed Framework for Functional Maturity Assessment

The preceding sections indicate that honey maturity is a continuous and multidimensional phenomenon. Therefore, no single parameter, whether morphological, microbiological, or physicochemical, is sufficient for its reliable assessment. Rather than treating honey as simply “mature” or “immature”, it is more appropriate to assess the degree of functional maturity in relation to microbiological stability, chemical quality, and fermentation risk. Natural variability related to botanical origin, climate, weather conditions, hive type, and beekeeping practices makes rigid universal classification difficult. However, the main quality and commercial risk associated with insufficient maturity, namely fermentation, can be assessed using measurable parameters, particularly water activity. The framework proposed in this section should be regarded as a conceptual and operational tool requiring further validation before possible use in official control practice.

### 7.1. Water Activity as a Priority Parameter

Among all the biomarkers mentioned, water activity ($a_{w}$) is proposed as the priority parameter for three reasons. First, it is a thermodynamic measure of the availability of water to microorganisms, not merely the water content per unit mass of product; it directly correlates with the biological possibility of osmophilic yeast growth, making it the only parameter that can replace the entire range of indirect microbiological indicators [10,11]. Second, measurement of $a_{w}$ is rapid (3–5 min), inexpensive, and can be performed at an apiary or control laboratory using a standard instrument such as AquaLab CX3TE or Rotronic HygroLab; the result is reproducible and unambiguous, requiring no expert interpretation [10]. Third, the criterion $a_{w}<0.60$ is based on a biologically justified mechanism—it is the physiological threshold of osmophilic yeasts determined in vitro under isobaric conditions [10,11]—and not on an arbitrarily chosen administrative value.

It is nevertheless necessary to conduct studies on whether, similarly to permitted exceptions regarding water content, exceptions should also be established in this regard. The microbiological stability of certain honey types may result not so much from low water activity as from specific components present in plant nectar.

### 7.2. Hierarchical Decision Framework

Based on the biomarkers described above, the proposed functional maturity assessment framework can be structured into two main analytical levels, organized according to the principle of proportionality. Level I provides basic screening based on moisture and water activity, whereas Level II provides an extended qualitative assessment based on enzymatic and chemical parameters for borderline or uncertain cases. Candidate metabolomic biomarkers are treated as a research-oriented validation layer requiring further multilaboratory verification rather than as routine official control parameters.

#### 7.2.1. Level I—Basic Screening

Level I constitutes a first-order filter based on two parameters measured directly on the product:

- Moisture ≤20% taking into account legally specified exceptions (refractometry, Codex STAN 12-1981 method), a normative parameter, mandatory in all major standards;
- Water activity $a_{w}<0.60$ taking into account possible exceptions, a parameter proposed as a normative supplement; when both of these criteria are met, the honey is microbiologically stable by virtue of the state of microbiological knowledge, and yeast growth is not observed and is effectively inhibited under standard storage conditions, regardless of their count [10,11].

Key rule of Level I: if moisture ≤ 20% AND $a_{w}<0.60$, the product is not subject to disqualification based on yeast count (CFU/g), regardless of the determined value of this parameter. Disqualification based solely on CFU/g, without confirmation of exceeding the $a_{w}$ threshold, is scientifically unjustified and legally inadequate [7,15].

If Level I indicates a borderline state (18.1–20.0% moisture or $a_{w}=0.60$–0.62) or the yeast count exceeds 1000 CFU/g, analysis is escalated to Level II.

#### 7.2.2. Level II—Extended Qualitative

**Figure 1:**
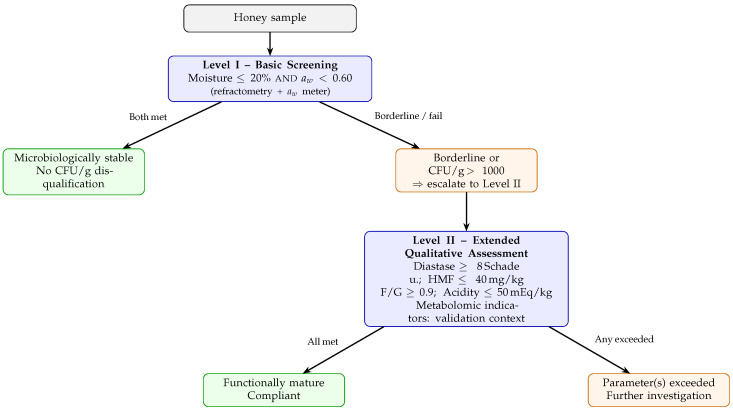
Hierarchical decision structure of the proposed functional maturity assessment framework (FMAM). Level I provides rapid basic screening based on moisture content and water activity $\left(a_{w}\right)$. Level II provides extended qualitative assessment for borderline or analytically uncertain cases using enzymatic and chemical parameters. Candidate metabolomic biomarkers are considered a research-oriented validation component requiring further multilaboratory verification. The framework is a conceptual model and should not be regarded as a validated official control procedure at this stage.

The hierarchical decision structure of the proposed functional maturity assessment framework, including the transition from basic screening to extended qualitative assessment, is summarized in Figure 1. Level II provides an assessment of functional maturity in all three dimensions defined in Section 3.3: microbiological, chemical, and biological stability. Level II parameters include:

1. Enzymatic profile:
  - Diastase activity ≥ 8 Schade units (or ≥3 units for botanical types with naturally low amylolytic activity, e.g., acacia, citrus, lavender) [7].
2. Chemical parameters:
  - F/G ratio ≥ 0.9; HMF ≤ 40 mg/kg; free acidity ≤ 50 mEq/kg (Codex) [7].

### 7.3. Recommendations for Regulatory Bodies and Apimondia

Based on the proposed FMAM and the conducted scientific analysis, this article formulates the following recommendations addressed to key stakeholders:

#### 7.3.1. Recommendations for Apimondia

1. Supplement the 2023 position with the $a_{w}$ criterion: the Apimondia recommendation should explicitly state that the presence of osmophilic yeasts in honey does not constitute grounds for declaring immaturity, provided moisture ≤ 20% and $a_{w}<0.60$; both parameters should be listed as simultaneous necessary conditions [10,11].
2. Take into account the regional diversity of production systems: the position should contain a clear clause regarding honeys from tropical climates and traditional systems, recognizing technological dehumidification as a legal production practice [23] or excluding specific dehumidification techniques that negatively affect the product.
3. Initiate dialogue with ISO TC 34/SC 17: the ISO 24607:2025 standard on bee honey should be extended to include the $a_{w}$ criterion as a normative parameter [7].

#### 7.3.2. Recommendations for EU Bodies (EC, DG SANTE)

Consider the inclusion of $a_{w}$ as a risk-based control parameter in official honey quality controls, particularly for consignments with borderline moisture values, products from high-humidity production systems, or cases in which fermentation risk is analytically uncertain.

#### 7.3.3. Recommendations for Laboratories and the Scientific Community

1. Standardize the multiparametric microbiological assessment protocol encompassing the simultaneous measurement of CFU/g, $a_{w}$, temperature, and phase state of honey (liquid/crystallized); results should be reported jointly, not as isolated values [10,27].
2. Perform multilaboratory validation of the decenedioic acid biomarker and extend its verification beyond three botanical types (rapeseed, acacia, and jujube) to tropical, stingless bee, and traditional production system honeys [14].
3. Develop portable in situ analysis tools: miniaturized NIR and Raman spectrometers allow measurement of moisture, $a_{w}$, and sugar profile directly at the apiary or at the border, with accuracy comparable to laboratory methods [20,46].

## 8. Conclusions and Future Perspectives

The conclusions of this review are presented in four complementary parts, covering the scientific interpretation of honey maturity, the regulatory implications of the Apimondia position, the proposed functional maturity assessment framework, and the limitations requiring further research.

This article undertook an analysis of the Apimondia position on the production and distribution of immature honey, with particular attention to the microbiological, regulatory, and market implications of using osmophilic yeast count and the morphological criterion of comb cell capping as quality indicators. The conducted analysis has led to a number of conclusions of varying degrees of generalization, from specific biochemical findings to normative recommendations of global scope.

### 8.1. Main Scientific Conclusions

(C1) The presence of osmophilic yeasts in honey is a biologically normal phenomenon. Yeasts of the genera *Zygosaccharomyces*, *Schizosaccharomyces*, and *Metschnikowia* are an inseparable component of the hive ecosystem, regardless of production method, hive system, and country of origin. The natural yeast population count in correct, non-fermenting commercial honey may amount to several hundred to a few thousand CFU/g without any effect on the microbiological stability of the product, provided water activity $a_{w}<0.60$ [11]. This conclusion has been well established in the literature for decades and confirmed by all current systematic reviews [8,21].

(C2) Water activity, not moisture content or yeast count, is the primary fermentation risk parameter. The physiological growth threshold of osmophilic yeasts ($a_{w}=0.61$–0.62) defines a biologically justified boundary below which honey fermentation is thermodynamically unfeasible and has not been observed under standard storage and temperature conditions, regardless of the yeast count expressed in CFU/g [10,11]. Glucose crystallization constitutes an additional factor that dynamically modifies the $a_{w}$ of the liquid phase, meaning that fermentation risk assessment must take into account the phase state of the product and cannot be based solely on the global water content [10,29].

(C3) Honey ripening is a continuous biological process, not a binary event. Maturity indicators—invertase, glucose oxidase, and diastase activity, organic acid profile, and fructose-to-glucose ratio—change gradually during the ripening process and do not correlate unambiguously with the moment of cell capping by bees [12,14]. Uncapped honey may exhibit a chemical profile appropriate for mature honey if moisture is low; capped honey may lose maturity characteristics as a result of inappropriate heat treatment [7,12,13].

(C4) Some honey maturity criteria are derived mainly from temperate-climate, frame-hive production systems and may have limited applicability under diverse global production and microclimatic conditions. The climatic, technological, and biological conditions of honey production in tropical and subtropical zones, including high environmental humidity, log and basket hive systems, and the distinct properties of stingless bee honeys (Meliponini), mean that uniform morphological (capping) and microbiological (CFU/g without $a_{w}$) criteria cannot serve as global quality indicators without systematically favoring honeys produced in European systems [23,36,37]. This position is consistent with the general trend in food legislation toward recognizing the diversity of local production systems and moving away from norms derived exclusively from Western European practices [31].

(C5) The dominant form of honey adulteration on the EU market is sugar syrup adulteration, not the distribution of honey with elevated yeast count. Data from the coordinated control action of the European Commission “From the Hives” (2021–2022) unambiguously indicate that 46% of suspect imported honey shipments contained additions of C_3_/C_4_ syrups or oligosaccharides [43]. None of the available control reports identifies elevated CFU/g count as the primary indicator of adulteration in inspection practice. Allocation of inspection resources toward $a_{w}$ measurement is therefore more appropriate to the actual market risk profile than the intensification of microbiological methods [20,43].

### 8.2. Regulatory and Practical Implications of the Apimondia Position

The Apimondia position of 2023 on immature honey is a valuable and necessary contribution to the protection of the integrity of the beekeeping market. The threat posed by systemically immature honey, produced by the method of industrial nectar dehydration in a short time and without maintaining the biological ripening processes, is real and constitutes unfair competition with producers adhering to quality standards. In this respect, the Apimondia position addresses an important and legitimate concern related to honey integrity and fair trade.

This article does not undermine this intention but indicates that the current formulation of the position is analytically incomplete in two important areas. First, it does not distinguish between (a) fermentation as a product characteristic at the time of harvest, and (b) fermentation as a post-harvest event resulting from supply chain errors. Second, it does not take into account the fundamental role of water activity as a parameter superior to yeast count in the assessment of fermentation risk. Supplementing the Apimondia position with these two elements will not weaken its protective message but will instead strengthen it scientifically and make it more resistant to legal challenges in trade disputes with importers from tropical countries [11,23,27].

Furthermore, an overly broad interpretation of yeast presence as an indicator of immaturity may lead to disproportionate assessment outcomes for honeys in which naturally elevated osmophilic yeast counts are frequent and not associated with active fermentation.

### 8.3. Functional Maturity Assessment Framework

The proposed functional maturity assessment framework (FMAM) offers a conceptual and operational basis for further development of honey quality assessment because it is:

- Scientifically justified, based on the thermodynamic properties of water activity, enzymatic ripening kinetics, and metabolomics [11,12,13];
- Compliant with applicable regulations—Codex STAN 12-1981 and EU Directive 2001/110/EC, and does not require their revision, only interpretive extension [7,15];
- Technically accessible, as $a_{w}$ measurement and refractometry can be performed in any control laboratory without specialized infrastructure [10,10];
- Potentially globally applicable, since criteria based on $a_{w}$ and enzymatic profile are independent of the hive system and climatic conditions of the place of production [23,37];
- Proportionate to the risk, preventing escalation to costly methods [20,24].

### 8.4. Limitations and Directions for Future Research

The authors of the article are aware of the limitations of this study. The proposed FMAM model is based on empirical data available at the time of manuscript preparation and requires further verification through prospective multilaboratory studies. The following directions for further research are particularly indicated:

1. Multilaboratory validation of the decenedioic acid biomarker on samples representing the global diversity of botanical types and production systems, extending beyond the three types (rapeseed, acacia, and jujube) studied by Sun et al. [14]; validation should encompass stingless bee honeys (Meliponini) and honeys from traditional East African systems [23].
2. Development and validation of a multidimensional functional maturity index (FMI, Functional Maturity Index) integrating the results of Level II parameters into a single index with a value of 0–100, calibrated for different botanical and geographical types; such an index could replace binary disqualification decisions with a gradient approach proportionate to the deviation from the optimal state [12,13,14].
3. Harmonization of ISO TC 34/SC 17 standards taking into account the meliponiculture production system—the stingless bee honey category requires a separate standardization track with its own moisture thresholds, $a_{w}$, and enzymatic criteria, taking into account the biological properties of these insects [37,38].

Undertaking this research is possible only within the framework of international cooperation encompassing scientific institutions, beekeeping organizations, and regulatory bodies from countries of both the “Global North” and tropical countries that are the main honey producers. This dialogue is not only a scientific need but a necessary condition for building a globally equitable and scientifically credible quality assessment system for one of the oldest food products manufactured by humankind.

## Figures and Tables

**Table 1:** Comparison of honey quality parameters in major international standards. Abbreviations: n.a., not applicable (no limit in the document); CFU, colony-forming units; HMF, hydroxymethylfurfural.

Standard	Moisture (%)	HMF (mg/kg)	Diastase (Schade u.)	CFU/g Limit Yeasts	Criterion Microbiolog.
Codex STAN 12-1981	≤20.0	≤40	≥8	none	prohibition of fermentation
EU Directive 2001/110/EC	≤20.0	≤40	≥8	none	prohibition of fermentation
USP (honey monograph)	≤21.0	n.a.	n.a.	none	prohibition of fermentation
Reg. (EC) 852/2004	n.a.	n.a.	n.a.	none	general food ^∗^

^∗^ The 100 CFU/g value may be used in some laboratory or inspection practices as a general hygiene indicator for yeasts and molds; however, it is not a honey-specific legal criterion established by Regulation (EC) No 852/2004.

**Table 2:** Estimated honey fermentation risk as a function of moisture and osmophilic yeast count (storage temperature 10–27 °C). Source: own compilation based on [27,30].

Moisture [%]	Yeast Count [CFU/g]		
	<10	10–1000	>1000
≤17.0	negligible risk	negligible risk	negligible risk
17.1–18.0	negligible risk	low risk	moderate risk
18.1–19.0	negligible risk	moderate risk	high risk
19.1–20.0	low risk	high risk	very high risk
>20.0	moderate risk	high risk	very high risk

**Table 3:** Comparison of beekeeping production systems with respect to honey maturity control. Source: own compilation based on [23,36,39].

Feature	Langstroth Hive	Kenya Top Bar Hive	Traditional Hive
Capping control	Full (frame inspection)	Partial	None (destructive)
In situ dehumidification	Yes	Limited	None
Average yield [kg/hive/year]	20–40	15–26 [39]	5–15 [23]
Typical honey moisture	16–19%	17–20%	17–23%
Risk of yeast colonization	Low	Moderate	High
Global use	Americas, Australia, Europe	East Africa	Africa, S.E. Asia

## Data Availability

No new data were created or analyzed in this study. Data sharing is not applicable to this article.
